# MELD-Na score may underestimate disease severity and risk of death in patients with metabolic dysfunction-associated steatotic liver disease (MASLD)

**DOI:** 10.1038/s41598-023-48819-6

**Published:** 2023-12-13

**Authors:** David Yardeni, Adi Shiloh, Inna Lipnizkiy, Anat Nevo-Shor, Naim Abufreha, Daniela Munteanu, Victor Novack, Ohad Etzion

**Affiliations:** 1 Department of Gastroenterology and Liver Diseases, Soroka University Medical Center, P.O.B. 151, 84101 Beersheba, Israel; 2 Clinical Research Center, Soroka University Medical Center, Beersheba, Israel

**Keywords:** Liver cirrhosis, Non-alcoholic fatty liver disease, Portal hypertension

## Abstract

Portal hypertension often precedes the development of advanced fibrosis in patients with Metabolic dysfunction-associated steatotic liver disease (MASLD) and may accelerate disease progression to cirrhosis. We aimed to evaluate whether prioritization tools accurately predict survival in patients with MASLD and clinically significant portal hypertension (CSPH). We retrospectively identified patients diagnosed with esophageal or gastric varices (EGV). Laboratory results, endoscopy reports and outcomes of patients with MASLD were compared to patients with advanced stage chronic liver disease (CLD) of other etiologies. During the study period 326 patients were diagnosed with EGV. 88 (26.9%) had MASLD, 113 (34.6%) viral hepatitis (VH), 63 (19.3%) alcoholic liver disease (ALD) and 62 (19%) both VH and ALD (VHALD). EGV bleeding events were significantly more frequent in patients with MASLD (36.3%), compared to VH (28.3%), ALD (30.1%) and VHALD (25.8%), respectively (p < 0.01). Mean Model for End-Stage Liver Disease (MELD)-Na score surrounding 1 year of first event of EGV bleeding was significantly lower in MASLD patients compared to all other etiologies (p = 0.02). At a MELD-Na score of 11–20, cumulative survival rate was significantly lower in MASLD patients compared to all other etiologies (log rank p < 0.01). MASLD patients present with EGV bleeding at lower MELD-Na scores compared to other etiologies of CLD. MELD-Na score may therefore underestimate disease severity and risk of death in patients with MASLD and CSPH.

## Introduction

Metabolic dysfunction-associated steatotic liver disease (MASLD) formerly known as non-alcoholic fatty liver disease (NAFLD) is a highly common liver pathology with a prevalence that may exceed 40% of the adult population in western and industrialized countries, with even higher rates among the obese and diabetic populations^1^. MASLD describes a clinical spectrum ranging from benign fat accumulation in hepatocytes, to fatty infiltration that is accompanied by inflammation and necrosis (steatohepatitis, MASH) ultimately leading to cirrhosis, hepatocellular carcinoma (HCC) and clinically significant portal hypertension (CSPH) with complications such as ascites and esophageal varices^2,3^. In recent years, cirrhosis and HCC due to MASLD have become an ever-growing indication for liver transplantation and this increased rate is expected to soon define it as the most common indication for liver transplantation^4^.

Esophageal varices are a potentially deadly complication of CSPH^5^. At least two-thirds of cirrhotic patients develop esophageal varices during their lifetime. It is generally thought that CSPH develops only after liver fibrosis has reached an advanced stage^6^. As the disruption of liver architecture caused by advanced fibrosis also leads to impairment in liver function, CSPH and decreased liver synthetic function seem to parallel each other. These observations are mainly based on studies conducted in patients with viral hepatitis. However, previous studies have shown that CSPH can precede the development of advanced fibrosis and cirrhosis in MASLD^7,8^. The mechanism underlying this phenomenon is presumed to be related to architectural changes such as steatosis and ballooning in the hepatic parenchyma which confer sinusoidal narrowing, with subsequent increase in hepatic venous pressure gradient (HVPG)^9,10^. Also, in MASH, fibrosis and ballooning begin around the central veins, possibly leading to central vein occlusion^11^. Other studies have shown complications of CSPH can occur at lower (< 10 mmHg) HVPG measurements than in viral hepatitis ^12,13^. Nakamura and colleagues have demonstrated that the incidence of esophageal or gastric varices (EGV) occurring in MASH patients is equivalent to or higher than in patients with chronic hepatitis C (HCV) and alcoholic liver disease (ALD)^14^.

During the last decade, the Mean Model for End-Stage Liver Disease (MELD) score and its modified heir, MELD-Na have become the standard patient prioritization tool on the liver transplant waiting list in the western world^15–19^. However, a major known limitation to the MELD score is underestimation of CSPH and related complications^20^.

In this study we set out to evaluate whether current prioritization for liver transplantation via the MELD-Na score accurately predicts survival in patients with MASLD and bleeding esophageal or gastric varices in comparison to patients with cirrhosis and CSPH due to other common etiologies of chronic liver disease (CLD). We focused our aim on variceal bleeding as it is a known harbinger of mortality in patients with CSPH^21^.

## Materials and methods

### Study population

We retrospectively investigated all patients who were hospitalized or visited the liver clinic at Soroka University Medical Center (SUMC) and presented with EGV due to advanced CLD from January 2010 to September 2019. SUMC is a 1200 bed academic center which serves as the only tertiary center for a population of over 1 million people living in the Negev region of Israel. Patients with a diagnosis of CLD were enrolled in the study if they were > 18 years of age and were diagnosed during esophagogastroduodenoscopy (EGD) with esophageal and/or gastric varices with or without bleeding. Patients were excluded from the study if they had known solid organ or hematological malignancy at the time of diagnosis or within 6 months of diagnosis. Patients were also excluded due to lack of sufficient data enabling conclusion of liver disease etiology or calculation of their MELD-Na score. MELD-Na score was calculated using average laboratory values 1 year before and after varices diagnosis at EGD and 1 year before and after first event of varices bleeding.

### Data sources and clinical definitions

All patients were identified by a national ID number and were members of Clalit Health Services (CHS), the largest health maintenance organization (HMO) in Israel. CHS maintains a computerized database, with complete records of patients’ medical history, laboratory and imaging test results, medications and mortality data. We collected all the data in the patient’s HMO computerized database from the day of enrollment until September 2019.

The following clinical data was recorded: demographics, body mass index (BMI), complete blood count, alanine aminotransferase (ALT), aspartate aminotransferase (AST), gamma glutamyl transferase (GGT), alkaline phosphatase (ALP), albumin, creatinine, Na, urea, bilirubin and International Normalized Ratio (INR). Etiology of CLD was determined by ICD-9 codes and corroborated by relevant virologic markers and imaging studies. Cases of cryptogenic cirrhosis were adjudicated to result from MASLD if review of individual patient records showed evidence suggestive of fatty liver on imaging studies and/or presence of comorbidities of the metabolic syndrome, and if other plausible causes of cirrhosis were ruled out. Presence of the following comorbidities was recorded: diabetes mellitus (DM)/impaired fasting glucose (IFG), dyslipidemia, hypertension (HTN), ischemic heart disease (IHD), chronic kidney disease (CKD). Endoscopic data regarding EGV grade, bleeding events and number of ligations performed was also recorded. Cause of in-hospital deaths, was manually retrieved from patient records. For patients who expired out-of-hospital, the primary diagnosis at their last admission to the hospital up to 3 months before expiration, was recorded as the attributable cause of death.

### Ethical statement

The study was approved by the institutional review board of SUMC and was conducted in compliance with the Declaration of Helsinki, Good Clinical Practice guidelines, and local regulatory requirements. The Institutional Review Board of SUMC (Soroka University Medical Center) for studies on existing patient data decided for request number 0378-19-SOR on a full waiver of informed consent that was given on September 12, 2019, for study protocol SCRC19029.

### Study outcomes

The primary hypothesis for the study was that patients with CLD due to MASLD suffer from liver related mortality at lower MELD-Na scores calculated near EGV diagnosis and compared to other underlying etiologies of CLD. The primary clinical outcome evaluated was 5-year survival following the diagnosis of EGV. Secondary outcomes included comparison of liver related and non-liver related causes of death, location and grade of EGV, frequency of bleeding events and changes in the MELD-Na score over time.

### Statistical analysis

When appropriate, univariate comparisons were made using χ^2^-test or Fisher's exact test for categorical variables and using analysis of variance (one-way ANOVA) or Kruskal–Wallis tests for quantitative variables. Survival curves were generated using the Kaplan–Meier method. Study groups were compared using the log rank test of significance to determine the significance of differences between survival curves. Multivariate cox regression models were used to estimate the survival differences between study groups while adjusting for potential confounding factors. In order to examine the association between study group and the rate of MELD-Na score progression over time, mixed model for repeated measures was performed. The model included group, time and baseline MELD-Na score as fixed covariates, as well as interaction between group and time and interaction between baseline MELD-Na score and time. Random intercepts accounted for the dependence in the repeated measures. A p-value of 0.05 or less (two-sided) was considered statistically significant. IBM SPSS software, version 25.0, was used for statistical analysis.

## Results

### Patient characteristics

**Figure 1:**
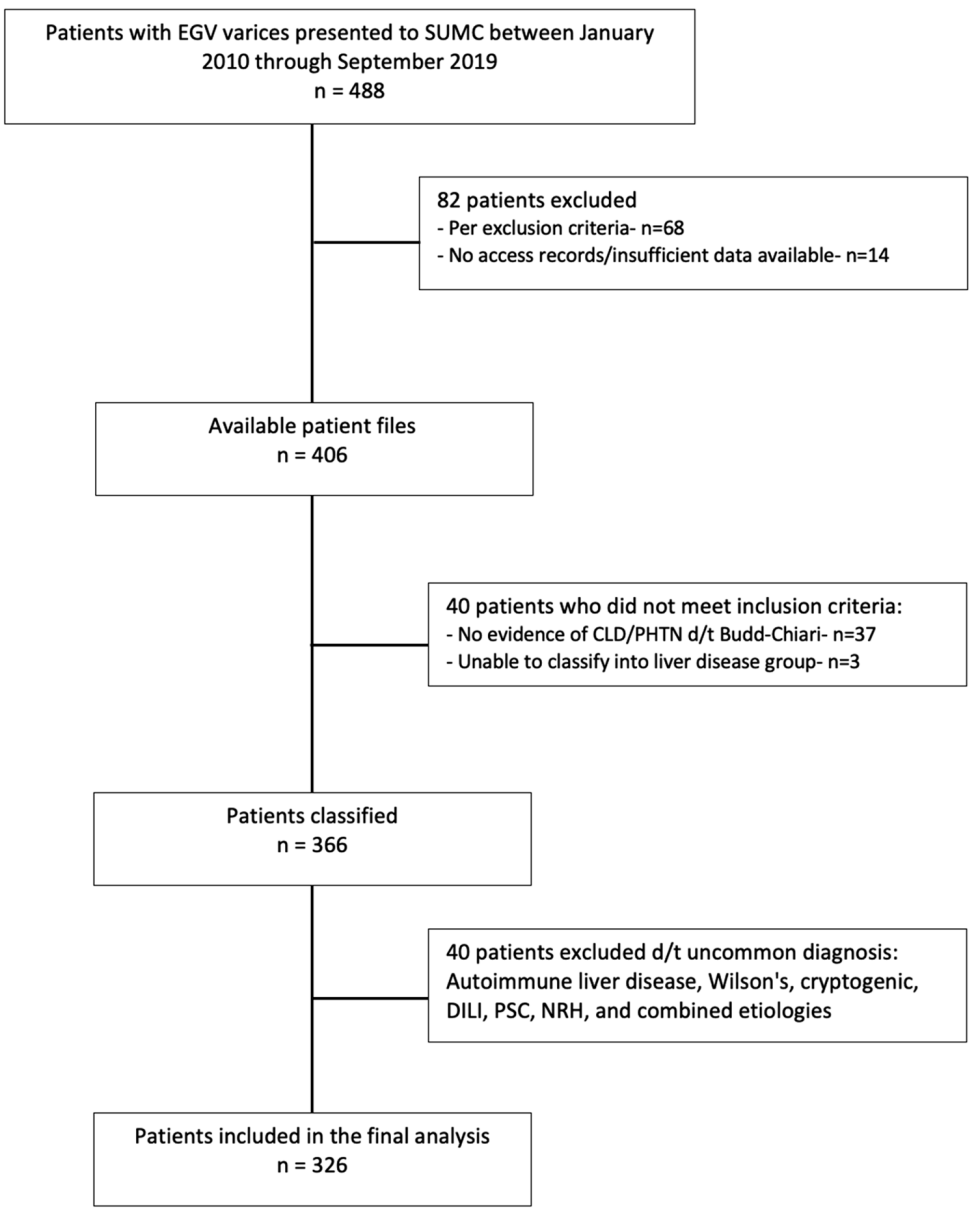
Study population flow chart. *EGV* esophageal or gastric varices, *CLD* chronic liver disease, *PHTN* portal hypertension, *DILI* drug induced liver injury, *PSC* primary sclerosing cholangitis, *NRH* nodular regenerative hyperplasia.

During the study period 488 patients were hospitalized or presented to the outpatient liver clinic at SUMC with EGV due to CLD (Fig. 1). 122 patients were excluded due to lack of evidence for a primary liver disease, vascular etiology of portal hypertension, or insufficient data to determine liver disease etiology or calculation of MELD-Na score. In addition, 40 patients were removed from the final analysis due to liver disease etiology of very low prevalence (e.g., Wilson’s disease). The final study cohort included 326 CLD patients (Table 1) who were classified into one of four groups: MASLD, 88 (26.9%); VH, 113 (34.6%); ALD, 63 (19.3%) and combined VH and ALD (VHALD), 62 (19%). Most study patients were males (210, 64.4%). Mean age was 60.8 ± 12.2 (years) and mean BMI was 29.1 ± 5.7 kg/m^2^. Most VH patients were infected by HCV (130, 74.3%), followed by chronic hepatitis B (HBV) (35, 20%) and a co-infection with HBV and hepatitis delta (5, 2.9%). Patients with MASLD were significantly older at EGV diagnosis compared to other etiologies of CLD (66.1 ± 10.7, p < 0.001). The MASLD group presented with higher rates of metabolic abnormalities and co-morbidities. BMI and Hemoglobin A1C% were significantly higher in MASLD patients compared to other groups (30.8 ± 5.3 kg/m^2^, p = 0.02 and 6.5 [5.8–7.8], p = 0.01, respectively). MASLD patients also displayed significantly higher rates of the metabolic comorbidities such as HTN, DM/IFG and dyslipidemia. IHD was also more prevalent among this group (27.3% (p = 0.01) compared to other etiologies of CLD. AST was significantly higher in the VHALD group at 76.6 U/L (47.8–111.6), p < 0.001. Significant differences were found in the mean values of ALT (p < 0.001), GGT (p < 0.001) and total bilirubin (p < 0.01) between the different patient groups. There were no significant differences in the peak achievable MELD-Na scores between patient groups up to 1 year before and after the diagnosis of EGV during EGD. At 1 year before and after varices diagnosis, the average MELD-Na score was 22 (13.5–26.5), 16 (12.8–28.8), 26 (21.5–36) and 29.5 (17–36.8) for MASLD, VH, ALD and VHALD, respectively (p = 0.18).

**Table 1:** Baseline characteristics of study participants.

	MASLD N = 88	Viral hepatitis N = 113	Alcoholic liver disease N = 63	Alcoholic liver disease and Viral hepatitis N = 62	p-value
Demographic characteristics					
Male sex, No. (%)	46 (52.3)	76 (67.3)	41 (65.1)	48 (77.4)	**0.01**
Age at varices diagnosis, mean ± SD	66.4 ± 10.7	60.5 ± 12	58.2 ± 13.2	56.3 ± 10.9	**< 0.001**
Jewish ethnicity, No. (%)	77 (87.5)	100 (88.5)	54 (85.7)	58 (93.5)	0.55
BMI, mean ± SD	30.8 ± 5.3	28.8 ± 5.4	28.8 ± 6.3	27.5 ± 5.8	**0.02**
DM/IFG, No. (%)	71 (80.7)	61 (54)	34 (54)	22 (35.5)	**< 0.001**
Dyslipidemia, No. (%)	53 (60.2)	30 (26.5)	32 (50.8)	10 (16.1)	**< 0.001**
HTN, No. (%)	55 (62.5)	53 (46.9)	33 (52.4)	24 (38.7)	**0.03**
IHD, No. (%)	24 (27.3)	21 (18.6)	8 (12.7)	4 (6.5)	**0.01**
CKD/renal failure, No. (%)	18 (20.5)	18 (15.9)	16 (25.4)	7 (11.3)	0.18
Lab results					
Hemoglobin	11.3 (9.6–12.9)	11.4 (10.2–13.3)	11.1 (9.5–12.9)	12.1 (10.3–14.1)	0.15
Platelets	101.2 (81.1–137.4)	87.6 (65.5–133.4)	107 (70.1–146.8)	91.7 (62.5–116.9)	0.21
INR	1.3 (1.1–1.5)	1.3 (1.1–1.5)	1.3 (1.2–1.7)	1.3 (1.1–1.7)	0.35
Creatinine	0.8 (0.7–1.1)	0.8 (0.7–1.1)	0.8 (0.6–1.2)	0.7 (0.6–1)	0.38
Urea	36.1 (28.6–46.3)	33.8 (26.5–48.5)	31.9 (26.5–48.8)	30.4 (23.8–37)	0.19
Albumin	3.4 (2.8–4)	3.4 (2.8–3.9)	3.3 (2.7–3.8)	3.3 (2.8–3.7)	0.4
AST	41.8 (32.2–54.8)	58.9 (41.6–90)	54.7 (37.4–91)	76.6 (47.8–111.6)	**< 0.001**
ALT	28 (19.1–38.7)	45.6 (29.9–67.7)	27.7 (21.3–56.5)	41.8 (28.1–59.1)	**< 0.001**
GGT	106.2 (56–170.7)	67.7 (36.2–121)	119.9 (75–256.6)	63.1 (43.9–168.6)	**< 0.001**
Bilirubin total	1.1 (0.8–1.7)	1.1 (0.8–2)	1.5 (0.9–2.8)	1.5 (1–3)	**< 0.01**
Alkaline phosphatase	114.1 (95.3–162.1)	107.4 (90.8–139.6)	121.9 (93.9–172)	115.2 (84–146.2)	0.23
Sodium	138.2 (136.8–140.1)	138.1 (136.5–140.3)	137.7 (135.1–140.8)	137.7 (136.3–139.8)	0.69
Hemoglobin A1C%	6.5 (5.8–7.8)	5.9 (5.2–7)	5.7 (5.2–6.6)	5.6 (5.1–7.2)	**0.01**
MELD-Na score*	22 (13.5–26.5)	16 (12.8–28.8)	26 (21.5–36)	29.5 (17–36.8)	0.18

*MELD* Model for end-stage liver disease, *MASLD* metabolic dysfunction-associated steatotic liver disease, *DM* diabetes mellitus, *IFG* impaired fasting glucose, *HTN* hypertension, *IHD* ischemic heart disease, *CKD* chronic kidney disease, *INR* international normalized ratio, *AST* aspartate aminotransferase, *ALT* alanine aminotransferase, *GGT* gamma glutamyl transferase.

*Peak MELD-Na score achievable 1 year before and after varices diagnosis at EGD, median (IQR).

Significant values are in bold.

### Clinical outcomes

Median follow-up duration was 54 months (29–78) for all patients and was similar between groups (Table 2). Overall, 152 (46.6%) patients expired during follow up. Mean age at death was 70 ± 11.7 for MASLD, 64.6 ± 11.7 for VH, 63.5 ± 10.6 for ALD, and 57.3 ± 11.6 for VHALD (P < 0.001). Five (4.4%) patients with VH and 2 (2.3%) with MASLD underwent liver transplantation (Supplementary Fig. 1) and 11 (3.37%) patients developed HCC during follow-up. There were no statistically significant differences in liver transplantation and HCC incidence between groups.

**Table 2:** Clinical measures and outcomes according to patient group.

	MASLD N = 88	Viral hepatitis N = 113	Alcoholic liver disease N = 63	Alcoholic liver disease and Viral hepatitis N = 62	p-value
Follow-up duration (months), median (IQR)	53 (28.3–77.3)	59 (33.5–80)	56 (23–79)	47 (26–74)	0.33
Deceased, No. (%)	49 (55.7)	49 (43.4)	30 (47.6)	24 (38.7)	0.17
Death age, mean ± SD	70 ± 11.7	64.6 ± 11.7	63.5 ± 10.6	57.3 ± 11.6	**< 0.001**
Months after varices diagnosis, median (IQR)	25 (9–45)	15 (1–38)	14.5 (3.8–29.3)	16.5 (5.3–25.8)	0.27
First year after varices diagnosis, No. (%)	14 (28.6)	23 (46.9)	14 (46.7)	9 (37.5)	0.23
Months after varices bleeding, median (IQR)	20 (1–38)	0.5 (0–22)	4 (0.5–19.5)	5 (0.8–21.8)	0.15
First year after varices bleeding, No. (%)	11 (47.8)	12 (66.7)	9 (69.2)	7 (70)	0.45
Transplanted, No. (%)	2 (2.3)	5 (4.4)	0 (0)	0 (0)	1 MASLD vs. other
Hepatocellular carcinoma, No. (%)	3 (3.4)	6 (5.3)	1 (1.6)	1 (1.6)	1 MASLD vs. other
MELD-Na score*	22 (16–26.5)	21 (14.8–31.3)	29 (25.5–38.5)	30.5 (18.5–39.8)	**0.01**
	22 (16–26.5)		26 (17–36)		**0.02** MASLD vs. other

*MELD* model for end-stage liver disease, *MASLD* metabolic dysfunction-associated steatotic liver disease.

*Peak MELD-Na score achievable 1 year before and after first event of varices bleeding, median (IQR).

Significant values are in bold.

Characteristics of EGV, bleeding and/or ligation events for all evaluated groups are presented in Table 3. No statistically significant differences were found in the presence and grade of EGV between groups throughout their follow up period. In addition, there was no significant difference between groups in the time from EGV diagnosis to the first bleeding event. Gastric varices were rare for all liver disease groups, with isolated gastric varices found in only 2 (2.3%) MASLD patients and 2 (1.8%) VH patients. Throughout the follow-up period, EGV Bleeding events were statistically significantly more frequent in patients with MASLD (36.3%), compared to VH (28.3%), ALD (30.1%) and VHALD (25.8%), respectively (p < 0.01). There were no differences in the rate of prescription of beta blockers between the different patient groups within 3 months of variceal bleeding (Supplementary Table 1). The peak achievable MELD-Na score (Table 2) during one year before and after the first EGV bleeding event was 22 (16–26.5), 21 (14.8–31.3), 29 (25.5–38.5), 30.5 (18.5–39.8) for MASLD, VH, ALD, VHALD, respectively (p = 0.01). For the MASLD group this score was significantly lower in comparison to all other CLD groups combined (p = 0.02).

**Table 3:** Varices characteristics at EGD according to patient group.

	MASLD N = 88	Viral hepatitis N = 113	Alcoholic liver disease N = 63	Alcoholic liver disease and Viral hepatitis N = 62	p-value
Maximum number of varices at one EGD, mean ± SD	3.6 ± 1.1	3.3 ± 1.3	3.5 ± 1.1	3.5 ± 0.9	0.41
Maximum number of band ligations applied at one EGD, mean ± SD	4.7 ± 1.7	4.8 ± 2.1	4.3 ± 1.7	4.5 ± 2	0.85
Varices grade, No. (%)					
Esophageal, grade 1	56 (63.6)	67 (59.3)	42 (66.7)	43 (69.4)	0.56
Esophageal, grade 2	52 (59.1)	54 (47.8)	31 (49.2)	31 (50)	0.42
Esophageal, grade 3	33 (37.5)	31 (27.4)	17 (27)	22 (35.5)	0.34
Gastroesophageal, grade 1	5 (5.7)	6 (5.3)	0 (0)	0 (0)	0.18 MASLD vs. other
Gastroesophageal, grade 2	1 (1.2)	2 (1.8)	0 (0)	0 (0)	1 MASLD vs. other
Isolated gastric, grade 1	2 (2.3)	2 (1.8)	0 (0)	0 (0)	0.3 MASLD vs. other
Bleeding and/or ligation events, No. (%)					**< 0.01 **MASLD vs. other
0	56 (63.6)	81 (71.7)	44 (69.8)	46 (74.2)	
1	16 (18.2)	19 (16.8)	9 (14.3)	6 (9.7)	
2–3	9 (10.2)	12 (10.6)	10 (15.9)	9 (14.5)	
4–5	7 (8)	1 (0.9)	0 (0)	1 (1.6)	
First bleeding event at time of varices diagnosis, No. (%)	18 (20.5)	17 (15)	11 (17.5)	7 (11.3)	0.48
Time from diagnosis to bleeding, for first bleeding event *after* the time of varices diagnosis (days) Median (IQR)	383 (69–745)	891 (399–1627)	560 (205–1322)	236 (102–632)	0.05

*MASLD* Metabolic dysfunction-associated steatotic Liver disease.

Significant values are in bold.

**Figure 2:**
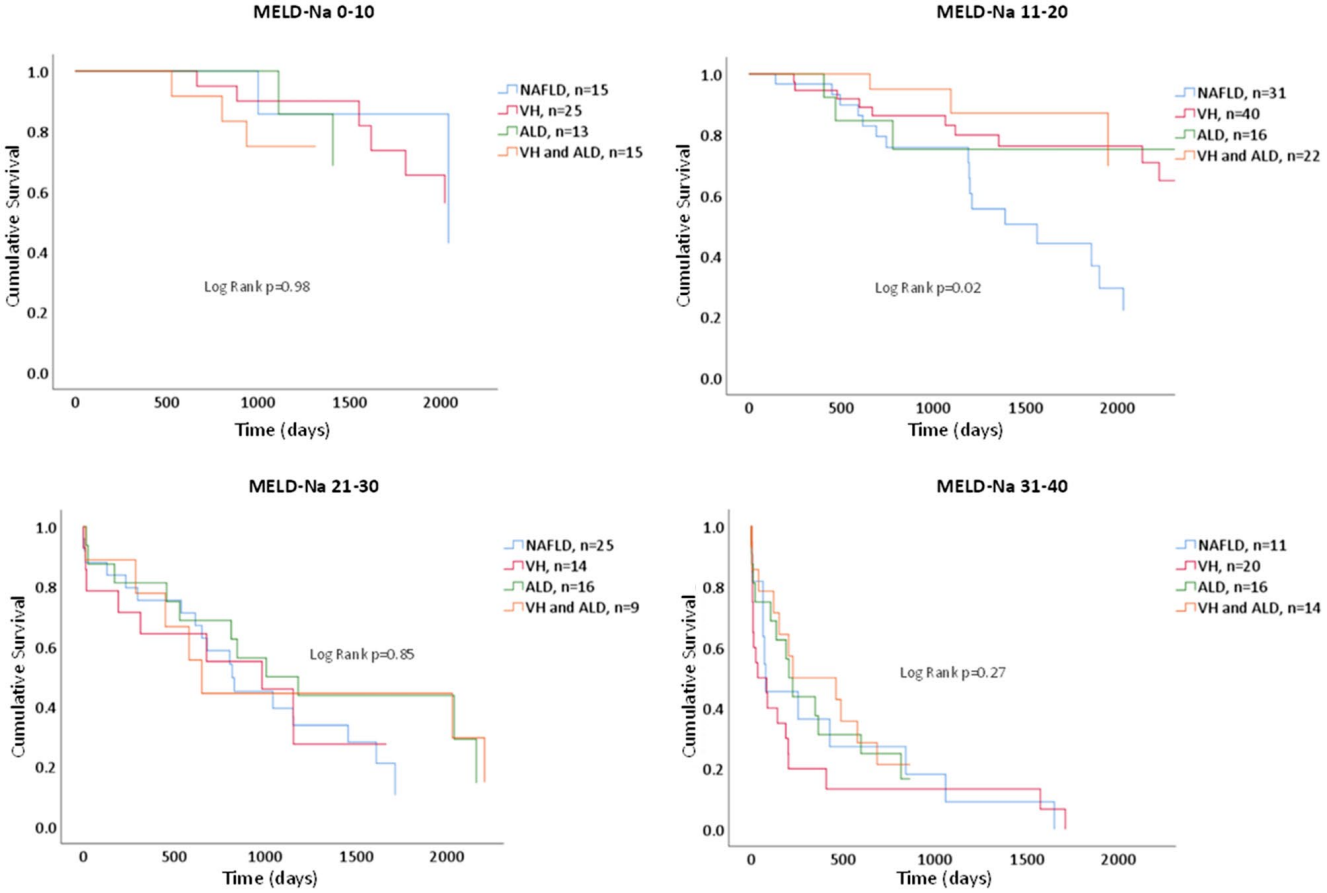
Kaplan–Meier curve of overall survival duration estimates for different patient groups following esophageal or gastric varices diagnosis stratified by *MELD-Na score range. *MELD-Na score calculated using peak achievable values one year before and after diagnosis of esophageal or gastric varices.

Overall cumulative survival rate for MASLD patients following EGV diagnosis was found to be significantly lower than all other CLD groups with hazard ratio (HR) calculated at 1.43 (1.01–2.01, p = 0.04). After adjustment for age, the difference became insignificant (p = 0.32). However, following adjustment for age, CKD, serum albumin and sodium, cumulative survival rate was once again significantly lower in the MASLD group, with a calculated HR of 1.67 (1.15–2.41, p < 0.01). When survival duration was stratified by peak MELD-Na score calculated around the time of EGV diagnosis, we found that compared to all other etiologies, patients with MASLD and an average MELD-Na score between 11 and 20, had statistically significantly lower cumulative survival rate, with HR calculated at 2.84 (1.42–5.65, p < 0.01) (Table 4, Fig. 2, Supplementary Fig. 2). Following utilization of the adjusted models, cumulative survival rate was lower in the MASLD group, with a calculated HR of 2.06 (0.97–4.41, p = 0.06). However, this difference did not achieve statistical significance. For a MELD-Na score range of 6–10, 21–30 and 31–40 no differences in survival were found in both adjusted and unadjusted models. When we evaluated the association between CLD etiology and repeated MELD-Na score measurements during follow-up, we found that the MELD-Na score progressed more rapidly in MASLD patients, with an average of 2.95 vs 1.17 point increase per year of MELD-Na score in the MASLD vs all other groups combined (Table 5, Supplementary Fig. 3).

**Table 4:** Cox regression analysis: association between liver disease etiology and survival, non-stratified and stratified by MELD-Na scores.

	Unadjusted HR (95% CI)	p-value	Model 1 HR (95% CI)	p-value	Model 2 HR (95% CI)	p-value
No stratification						
ALD, VH and both (reference)						
MASLD	1.43 (1.01–2.01)	0.04	1.2 (0.84–1.72)	0.32	1.67 (1.15–2.41)	< 0.01
MELD-Na 6–10						
ALD, VH and both (reference)						
MASLD	1.01 (0.22–4.59)	0.99	1.02 (0.96–4.37)	0.94	1.49 (0.31–7.26)	0.61
MELD-Na 11–20						
ALD, VH and both (reference)						
MASLD	2.84 (1.42–5.65)	**< 0.01**	1.99 (0.94–4.20)	0.07	2.06 (0.97–4.41)	0.06
MELD-Na 21–30						
ALD, VH and both (reference)						
MASLD	1.30 (0.71–2.38)	0.40	1.03 (0.54–1.96)	0.94	1.32 (0.65–2.66)	0.44
MELD-Na 31–40						
ALD, VH and both (reference)						
MASLD	1.08 (0.56–2.11)	0.82	1.02 (0.52–1.98)	0.97	1.43 (0.70–2.94)	0.33

Model 1: adjusted for age.

Model 2: adjusted for age, CKD, serum albumin and sodium.

*HR* hazard ratio, *MELD* model for end-stage liver disease, *ALD* alcoholic liver disease, *VH* viral hepatitis, *MASLD* metabolic dysfunction-associated steatotic liver disease.

Significant values are in bold.

**Table 5:** Association between liver disease and repeated measures of MELD-Na score over 6 years of follow-up.

	Estimate (95% CI)	p-value
MASLD vs. other	− 1.86 (− 3.47 to 0.25)	0.02
Time	1.17 (0.32–2.52)	0.008
Group*time	1.78 (1.05–2.52)	< 0.001
Baseline MELD-Na score	1.24 (1.10–1.37)	< 0.001
Baseline MELD-Na score*time	− 0.09 (− 0.17 to 0.02)	0.01

Obtained with multilevel mixed models with intercept as random effects.

*MELD* model for end-stage liver disease, *MASLD* metabolic dysfunction-associated steatotic liver disease.

During the follow-up period 49 (55.7%) patients with MASLD and 103 (43.2%) patients with CLD from other etiologies expired (Table 6, Supplementary Fig. 4). For both MASLD patients and non-MASLD patients, CLD and CSPH complications including gastrointestinal bleeding (GIB), Spontaneous bacterial peritonitis (SBP), acute on chronic liver failure (ACLF), hepatorenal syndrome (HRS) and HCC were found to be the most common cause of death at 67.3% and 59.2% respectively. Bleeding from EGV was the direct cause of death in 6 (12.2%) patients with MASLD and 18 (17.4%) patients with CLD from other etiologies (p = 0.49%). No significant differences were found regarding the cause of death between groups. Interestingly, cardiovascular disease (CVD) complications were responsible for only 12.2% of deaths in the MASLD group.

**Table 6:** Cause of death in patients with MASLD compared to other chronic liver diseases combined, No. (%).

	MASLD N = 49	Other liver diseases N = 103	p-value
Portal hypertension related complications	33 (67.3)	61 (59.2)	0.34
Infection—non liver related	7 (14.3)	21 (20.4)	0.36
Extrahepatic malignancy	1 (2)	4 (3.9)	1
Cardiovascular	6 (12.2)	5 (4.9)	0.18
Other	0 (0)	6 (5.8)	0.18
Unknown	2 (4.1)	6 (5.8)	1

Portal hypertension related complications: gastrointestinal bleeding; spontaneous bacterial peritonitis; acute on chronic liver failure; hepatorenal syndrome; hepatocellular carcinoma.

## Discussion

In this retrospective analysis of patients with CLD and endoscopic diagnosis of EGV we have found that MASLD patients present at lower MELD-Na scores surrounding the first event of EGV bleeding, compared to other major etiologies of CLD. In addition, patients with MASLD had a significantly higher number of EGV bleeding events compared to all other etiologies combined. In addition, we were able to show a trend for reduced overall survival of MASLD patients with EGV and specifically patients in the calculated MELD-Na score range of 11–20, in comparison to patients suffering from other causes of CLD. We have also shown that in our MASLD patient group the MELD-Na score progressed at a faster rate than observed in the other groups. Finally, our analysis revealed that the main causes for mortality in our MASLD patient group were liver related. It is therefore possible to conclude from our study that MELD-Na score may underestimate liver disease severity and risk of death in patients with MASLD and CSPH.

The MASLD patient group evaluated in our study was undoubtfully older and was found to have higher BMI levels as compared to the VH, ALD and VHALD group. However, even after adjusting and correcting our survival model, we noticed a reduced survival rate (nearly significant at p = 0.06) in MASLD patients with a MELD-Na score of 11–20. The reason for an increased mortality trend in this specific group is not entirely clear. One possible explanation is that patients are usually listed for transplantation at this MELD-Na range, and therefore a lower score within this range in MASLD patients with more pronounced CSPH, might expose them to significant morbidity contributing to their increased risk of death. This hypothesis is supported by the observation of increased number of EGV bleeding episodes in MASLD patients vs other groups. The reason why no significant or near significant differences in mortality rates were seen between patient groups in other MELD-Na score ranges may be explained by lack of severe disease burden in patients with MELD-Na of 6–10, which translates into a rather “flat”, low mortality rate for all patient groups. On the contrary, at MELD-Na ranges of 21–30 and 31–40 disease progression to significant impairment of synthetic function and accelerated elevation in portal pressure, impact MASLD and other patient groups alike, therefore leading to similar mortality rates.

The widespread use of the MELD score has completely changed liver transplant waiting list time and prioritization of organ allocation to patients^15–19^. No longer allocating patients by their length of time on the waiting list or by the semi-subjective Child-Turcotte-Pugh (CTP) classification, implementation of the MELD score in the United States (US) decreased mortality on the waiting list by 12% within a year^22^. However, several short comings of the MELD score are already well known^23^. Biases include reduced scores for females due to lower creatinine levels resulting from a lower muscle mass and an obvious lack of appreciation for ascites and hepatic encephalopathy which were both major components of the CTP evaluation. Much like ascites and hepatic encephalopathy, bleeding from EGV is another dreaded complication of CSPH that is not properly reflected by MELD or MELD-Na score. An attempt to integrate von Willebrand factor (vWF) into the MELD-Na score as a parameter of CSPH was recently attempted but needs further validation^24,25^. However, the currently available studies harnessing vWF for CSPH appreciation already suggest this integration can possibly improve organ allocation and thus further reduce mortality on the waiting list.

Furthermore, in the case of MASLD, it seems prediction of waiting list mortality using the MELD-Na score is far more problematic. The very nature of MASLD pathophysiology that begins with hepatic steatosis has the potential to alter the very delicate architecture of the hepatic parenchyma and thus induce an increase in the HVPG^9,10^. As this initial insult takes place prior to hepatic decompensation and synthetic abnormalities, manifestations of CSPH will already be dominating the patient’s clinical presentation and increasing the risk of mortality without a concomitant increase in the MELD-Na score. Such a discrepancy between severity of CSPH and the calculated MELD-Na score is only second to the one seen in patients suffering from non-cirrhotic portal hypertension (NCPH)^26^. Unfortunately, unlike NCPH, MASLD is an extremely common condition that already affects an ever-growing percentage of the world population and is soon to become the principal indication for liver transplantation in the US and other countries ^1^. This prospect is expected to generate a growing population of advanced MASLD patients with CSPH complications (such as bleeding EGV) and increased risk of death that is not adequately reflected by relatively low MELD-Na scores. In this setting, continued use of MELD-Na as the preferred system for organ allocation may lead to sub-optimal prioritization of MASLD patients for receiving liver transplant, while awaiting on the MELD-Na generated transplant waiting list. Interestingly, the recently developed MELD 3.0 was reported to better predict waitlist mortality by incorporating serum creatinine, serum albumin and female sex^27^. However, the effect of MELD 3.0 on the organ allocation rate for the ever-growing population of MASLD patients remains to be seen^1,28^.

Previous population-based studies have suggested CVD to be the leading cause of death in MASLD patients^29,30^. However, a recent prospective study from the NASH CRN group which followed 1773 adults with MASLD found an increased risk of mostly liver-related complications and death in patients progressing towards advanced liver fibrosis^31^. Similarly, the main cause of death in our cirrhotic MASLD patient group was found to be liver-related complications. In fact, 67.3% of mortality cases in our patient group were directly related to either liver failure, liver neoplasia and portal hypertension related complications. In contrast, CVD was found to be the cause of death in only 12.2% of the patients. The elucidation of mortality causes in our patient group further supports our hypothesis regarding underestimation of disease severity and the risk of death while on the waiting list for MASLD patients by the MELD-Na score.

The main strength of our study is the validated long term outcome data for each patient evaluated in our cohort. The high retention rate of patients evaluated at SUMC enabled us to accurately capture outcomes for any given patient presenting to the liver clinic with EGV. Furthermore, SUMC EMR database is also linked to CHS primary care clinics data sources and therefore allows continuity in data tracing of patients between community and hospital settings. Thus, for all patients included, the data collected allowed delineation of underlying liver disease etiology and elucidation of the probable cause of death of all MASLD patients in the study, but two.

Our study also presents several limitations including its retrospective design and the fact that data collection was limited to patients who underwent endoscopic evaluation at our institution. It is therefore possible that patients evaluated in an outside clinic were excluded from analysis. Another important limitation to our study, linked to its retrospective design, is the heterogenicity of patients with advanced CLD observed in the study. Grouping the non-MASLD patients into VH, ALD and VHALD groups provided us with robust data. However, it is possible that the different disease courses of VH (suppressed or cured HBV/HCV) and ALD (possible ongoing drinking) could have affected our study outcomes. Furthermore, it is important to mention that data collection for our study was performed prior to the 2023 consensus statement on fatty liver disease nomenclature. Therefore, a possible misclassification of patients with MetALD (MASLD and increased alcohol intake) as ALD or vice versa could have occurred. In addition, due to missing data during variceal bleed hospital admissions, we utilized an analysis of the peak achievable MELD-Na score at 1 year before and after the event as opposed to comparing direct measurable data from the index bleeding event. Finally, we report observations from a single center study that included only 326 patients in its final analysis. While findings from this study are novel and challenge current paradigms regarding MELD-Na system validity in advanced stage MASLD, they should be interpreted within the limits of the study design and scale. A multi-center prospective study allowing long term follow-up of a large population of patients with advanced MASLD may provide further insight on the impact of EGV bleeding on clinical outcomes and survival and on the ability of the MELD-Na or the MELD 3.0 score to predict it.

In conclusion, in this retrospective analysis of cirrhotic patients presenting with EGV, we have shown that MASLD patients can develop complications of CSPH such as variceal bleeding at lower MELD-Na scores compared to other etiologies of CLD. This can potentially result in excess mortality from liver related complications while on the liver transplant waiting list. We believe that development of novel prioritization tools for liver transplantation that incorporate portal hypertension severity measures should be encouraged and further validated in patients with MASLD and advanced liver disease.

### Supplementary Information

Supplementary Information.

## Data Availability

The datasets used and/or analyzed during the current study are available from the corresponding author upon reasonable request.

## Abbreviations

MASLD: Metabolic dysfunction-associated steatotic liver disease
NAFLD: Non-alcoholic fatty liver disease
MASH: Metabolic dysfunction-associated steatohepatitis
HCC: Hepatocellular carcinoma
CSPH: Clinically significant portal hypertension
HVPG: Hepatic venous pressure gradient
EGV: Esophageal or gastric varices
HCV: Hepatitis C virus
ALD: Alcoholic liver disease
MELD: Model for end stage liver disease
CLD: Chronic liver disease
EGD: Esophagogastroduodenoscopy
BMI: Body mass index
ALT: Alanine aminotransferase
AST: Aspartate aminotransferase
GGT: Gamma glutamyl transferase
ALP: Alkaline phosphatase
INR: International normalized ratio
DM: Diabetes mellitus
IFG: Impaired fasting glucose
HTN: Hypertension
IHD: Ischemic heart disease
CKD: Chronic kidney disease
VH: Viral hepatitis
VHALD: Viral hepatitis and alcoholic liver disease
NCPH: Non-cirrhotic portal hypertension
HR: Hazard ratio
CVD: Cardiovascular disease
