# Supplementary material for: MELD-Na score may underestimate disease severity and risk of death in patients with metabolic dysfunction-associated steatotic liver disease (MASLD)
Source: Sci Rep. 2023 Dec 13;13:22113. doi: 10.1038/s41598-023-48819-6 (PMC10719367; doi:10.1038/s41598-023-48819-6)
Supplement: Supplementary file 1 — Supplementary Information. [file 41598_2023_48819_MOESM1_ESM.docx]

**MELD Score May Underestimate Disease Severity and Risk of Death in Patients with Metabolic dysfunction-associated steatotic liver disease (MASLD)**

David Yardeni^1^, Adi Shiloh^2^, Inna Lipnizkiy^1^, Anat Nevo-Shor^1^, Naim Abufreha^1^, Daniela Munteanu^1^, Victor Novack^2^, Ohad Etzion^1^

1. *Department of Gastroenterology and Liver Diseases, Soroka University Medical Center, Beersheba, Israel.*
2. *Clinical Research Center, Soroka University Medical Center, Beersheba, Israel.*

**Table of Contents:**

Page 2 Supplementary Table 1

Page 3 Supplementary Figure 1

Page 4 Supplementary Figure 2

Page 5 Supplementary Figure 3

Page 6 Supplementary Figure 4

| **Supplementary Table 1:** Beta Blockers treatment | | | | | |
| --- | --- | --- | --- | --- | --- |
|  | **MASLD**  **N=32** | **Viral hepatitis**  **N=32** | **Alcoholic liver disease**  **N=19** | **Alcoholic liver disease and Viral hepatitis N=16** | **P-value** |
| Beta blockers treatment around first event of variceal bleeding* | 24 (75) | 22 (68.8) | 17 (89.5) | 14 (87.5) |  |
|  | 24 (75) | 53 (79.1) | | | 0.65 |
| Beta blockers purchased within 3 months of varices diagnosis or bleeding  * Among patients who had at least one event of variceal bleeding  MASLD, metabolic dysfunction-associated steatotic liver disease | | | | | |

**Supplementary Figure 1:** Liver transplantation patients characteristics

Patients who have undergone liver transplantation

n = 22

Liver transplantation was performed after EGV documentation

n = 16

Patients available for classification

n = 14

Patients were excluded due to diagnosis of malignancy within 6 months of EGV diagnosis

n = 1

Cases included in the analysis

n = 7

Other; Autoimmune liver disease (6), Combined etiologies (1)

n = 7

Liver transplantation was performed prior to EGV documentation

n = 6

Not enough data available

n = 1

MASLD

n = 2

Viral hepatitis; HBV (3), HCV (1), HBV and HDV (1)

n = 5

**Supplementary Figure 2**: Kaplan-Meier overall survival duration estimates for MASLD patients and all other ESLD patients with gastroesophageal varices following varices diagnosis and different MELD-Na scores calculated 1 year around varices diagnosis


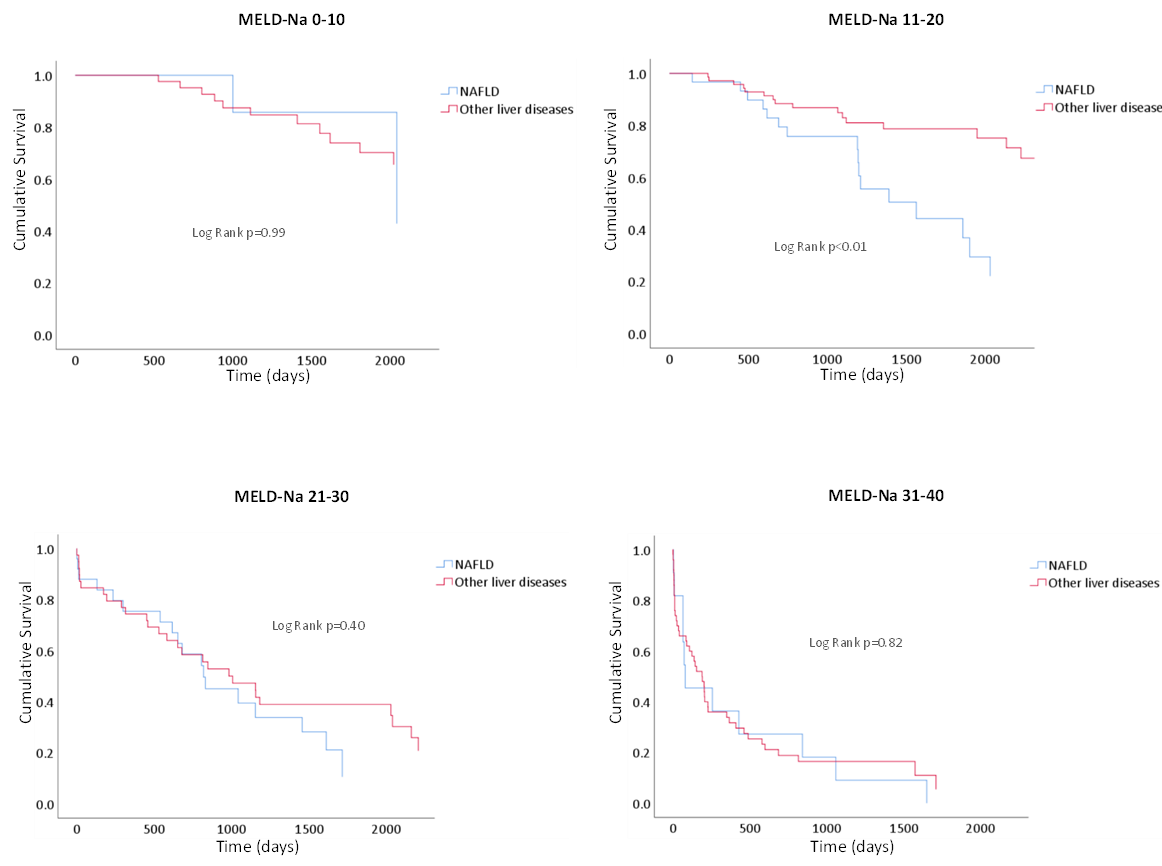


**Supplementary Figure 3:** MELD-Na score progression over 6 years of follow-up since the diagnosis of GEV, presented as change from baseline

MELD – model for end-stage liver disease; MASLD – metabolic dysfunction-associated steatotic liver disease; VH – viral hepatitis; ALD – alcoholic liver disease


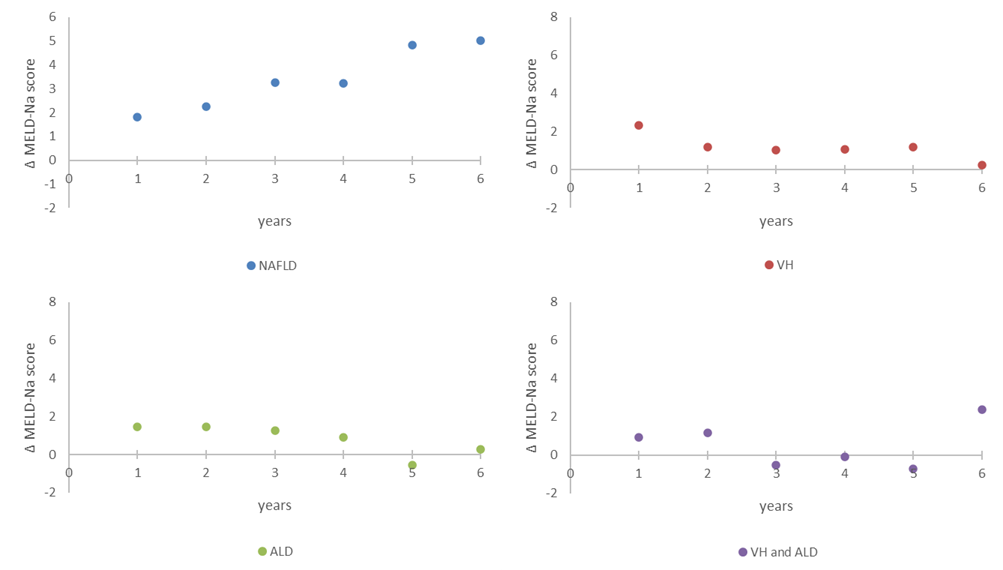


**Supplementary Figure 4:** Cause of death of patients with non-alcoholic fatty liver disease and CLD from other reasons

GIB – gastrointestinal bleeding; SBP – spontaneous bacterial peritonitis; ACLF – acute on chronic liver failure; HRS – hepatorenal syndrome; HCC – hepatocellular carcinoma


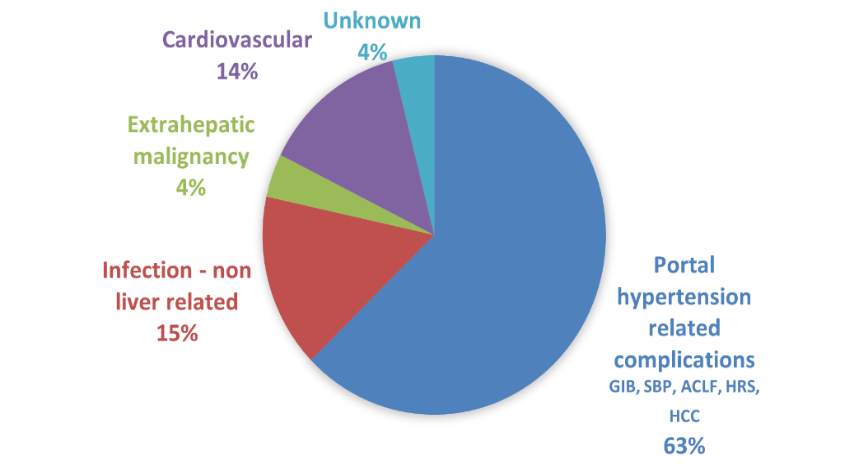

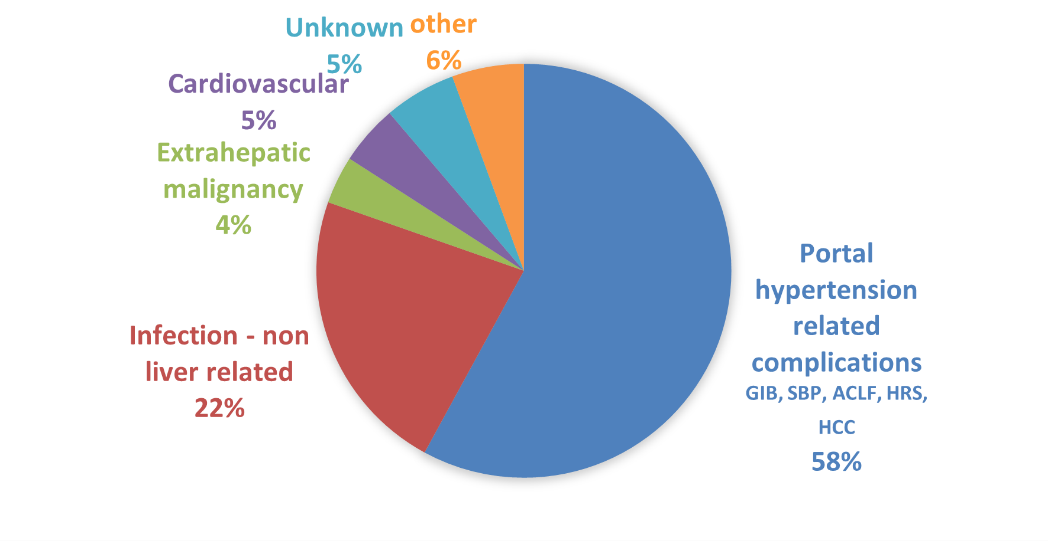


MASLD

Other liver diseases
